# Protective efficacy of combined use of parecoxib and dexmedetomidine on postoperative hyperalgesia and early cognitive dysfunction after laparoscopic cholecystectomy for elderly patients^1^

**DOI:** 10.1590/s0102-865020190090000005

**Published:** 2019-11-28

**Authors:** Xiaoxuan Du, Feng Song, Xueqiang Zhang, Shanshan Ma

**Affiliations:** I MD, Department of Anesthesiology, the Sixth Affiliated Hospital of the Sixth Clinical Medical School, Xinjiang Medical University, Tianshan District, Urumqi, XinJiang, China. Design of the study, technical procedures, manuscript writing; II MD, Department of Anesthesiology, the Sixth Affiliated Hospital of the Sixth Clinical Medical School, Xinjiang Medical University, Tianshan District, Urumqi, XinJiang, China. Technical procedures, analysis of data, critical revision; III MM, Department of Anesthesiology, the Sixth Affiliated Hospital of the Sixth Clinical Medical School, Xinjiang Medical University, Tianshan District, Urumqi, XinJiang, China. Technical procedures, analysis of data, critical revision

**Keywords:** Dexmedetomidine, Pain, Cholecystectomy, Laparoscopic

## Abstract

**Purpose:**

To investigate efficacy of combined use of parecoxib and dexmedetomidine on postoperative pain and early cognitive dysfunction after laparoscopic cholecystectomy for elderly patients.

**Methods:**

The present prospective randomized controlled study included a total of 80 patients who underwent laparoscopic cholecystectomy surgery during January 2016 to November 2017 in our hospital. All patients were randomly divided into 4 groups, the parecoxib group, the dexmedetomidine group, the parecoxib and dexmedetomidine combined group, and the control group. Demographic data and clinical data were collected. Indexes of heart rate (HR), mean arterial pressure (MAP), levels of jugular venous oxygen saturation (SjvO2) and jugular venous oxygen pressure (PjvO2) were recorded at different time points before and during the surgery. The mini-mental state examination (MMSE) score, Ramsay score and Visual Analogue Score (VAS) were measured.

**Results:**

Levels of both SjvO2 and PjvO2 were significantly higher in parecoxib group, dexmedetomidine group and the combined group than the control group. Meanwhile, levels of both SjvO2 and PjvO2 in the combined group were the highest. VAS scores were significantly lower in the combined group than all other groups, and total patient controlled intravenous analgesia (PCIA) pressing times within 48 h after surgery were the lowest in the combined group. Both Ramsay and MMSE scores were the highest in the combined group compared with other groups, while were the lowest in the control group.

**Conclusion:**

The combined use of parecoxib and dexmedetomidine could reduce the postoperative pain and improve the postoperative sedation and cognitive conditions of patients after laparoscopic cholecystectomy.

## Introduction

Laparoscopic cholecystectomy is widely accepted as the standard operation for benign gallbladder disease^1^, including gallstones^2^, acute or chronic cholecystitis^3^ ^,^ ^4^ and biliary pancreatitis^5^. Despite the well-known advantages of laparoscopic cholecystectomy compared to open cholecystectomy, such as being a safer, more effective procedure, with less complications^6^, the postoperative pain and cognitive dysfunction (POCD) are still two major problems influencing patients' recovery^7^ ^,^ ^8^.

Parecoxib, a highly selective COX-2 inhibitor, is reported to be used in the control of postoperative pain^9^ ^,^ ^10^. Studies show that Parecoxib could be used in postoperative pain control in many surgeries such as laparoscopic surgeries^11^, total hip arthroplasty^12^, and cancer related operations^13^. Dexmedetomidine (DEX), a kind of α2- adrenergic receptor agonist with sedative, analgesic, and anxiolytic properties has been applied for operative anesthesia, postoperative care, especially in mechanical ventilation and/or sedation-dependent procedures as a sole sedative or as an adjunct drug. Previous studies showed that dexmedetomidine can be used in surgery of laparoscopic cholecystectomy and could reduce postoperative pain^14^. However, to our best knowledge, few studies focused on efficacy of the combined use of parecoxib and dexmedetomidine on postoperative pain and early cognitive dysfunction after laparoscopic cholecystectomy for elderly patients.

In the present study, we aimed to investigate whether the combined use of parecoxib and dexmedetomidine could improve postoperative pain and early cognitive dysfunction after laparoscopic cholecystectomy for elderly patients. This study might give more clinical evidence for the application of parecoxib and dexmedetomidine in postoperative treatment of elderly patients under laparoscopic cholecystectomy.

## Methods

The present study was approved by the ethics committee of the Sixth Affiliated Hospital of the Sixth Clinical Medical School of Xinjiang Medical University.

This prospective randomized controlled single blinded study included a total of 80 patients who underwent laparoscopic cholecystectomy surgery from January 2016 to November 2017 in our hospital. All the patients who met the inclusion criteria were consecutively enrolled during the period. All patients were diagnosed as grade I~II according to the American Association of Anesthesiologists (ASA) and were older than 65 years with a mean age of 69.2±4.3 year. The following patients were excluded: patients with preoperative cognitive dysfunction; patients with psychosis; patients with severe cardiac, lung, liver, renal or other system diseases. Patients were also excluded if the surgery time was longer than 1h, or the surgery could not be accomplished successfully and turned to laparotomy operation. Informed consent was obtained from all patients.

### Treatment and analgesia strategies

All patients were randomly divided into 4 groups with 20 cases in each according to a computer generated randomization list run by the hospital pharmacist, 1) the parecoxib group, with pre-intravenous injection of 40 mg parecoxib 30 min before the surgery; 2) the dexmedetomidine group, in which the patients were treated with 0.6 μg/kg/h dexmedetomidine by intravenous injection after anesthesia induction to the end of the surgery; 3) the combined-group, in which patients were treated with both pre-intravenous injection of 40 mg parecoxib and 0.6 μg/kg/h dexmedetomidine by intravenous injection after anesthesia induction to the end of the surgery; 4) the control group, in which patients were treated with equal amount of physiological saline. The surgery strategy for all patients followed the same protocol and was conducted by the same team.

For analgesia strategies, the pre-treatment of parecoxib was performed by pre- intravenous injection with 100 ml normal saline with 40 mg parecoxib^15^. The analgesia induction was conducted by intravenous injection of 1~2 mg/kg propofol, 0.15 mg/kg cisatracurium and 2-4 μg/kg fentanyl citrate followed with endotracheal intubation and mechanical ventilation. For all groups, the anesthesia maintenance was conducted using sevoflurane inhalation anesthesia. For the dexmedetomidine and the combined group, 0.6 μg/kg/h dexmedetomidine was kept by intravenous injection until the end of the surgery. For the control group, 0.9% NaCl was given under 0.6 μg/kg/h. The postoperative patient-controlled intravenous analgesia pump (PCIA) was used for all patients. The constitution of drugs in patient-controlled intravenous analgesia pump (automatic electronic drug injection pump ZZB- II type, Jiangsu AI Peng Medical Equipment Co., Ltd., China) was: tartaric acid Bhutto 6mg + sufentanil citrate 0.02ug/kg/h + 0.9% physiological saline 100ml.

### Data collection and measurement

Demographic data like age, gender, body mass index (BMI), and clinical data including ASA stage, pathological type and mean operative time were collected. Indexes of heart rate (HR) and mean arterial pressure (MAP) were all recorded. Levels of jugular venous oxygen saturation (SjvO2) and jugular venous oxygen pressure (PjvO2) were measured by blood gas analysis using a blood gas analyzer (GEM Premier3000, Instrumentation Laboratory, MA, USA) at different time points as follows: T0 entering the surgery room, T1 trachea cannula, T2 tracheal extubation, T3 10 min after tracheal extubation. The mini-mental state examination (MMSE) score and Ramsay score were measured at 12 h, 24 h, and 48 h after surgery and Visual Analogue Score (VAS) was measured at 4 h, 12 h, 24 h, and 48 h after surgery. The PCIA pressing time within 48 h and complications within 3 months after surgery were also recorded.

### Statistical analysis

The measurement data was expressed by mean ± SD. Counting materials were compared using Chi square test. Comparisons among three or more groups were conducted using one-way analysis of variance (ANOVA) followed by Tukey post hoc test. It was considered to be statistically significant when *P*-value was less than 0.05. All calculations were made using SPSS 20.0.

## Results

### Basic clinical information for all patients

**Figure 1:**
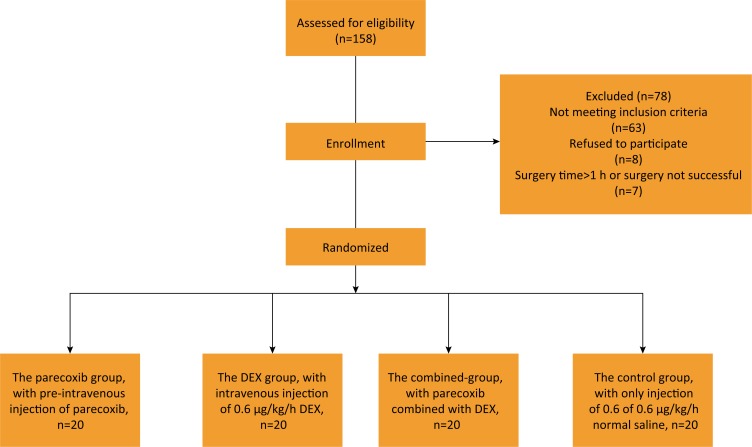
Flow chart of the present study.

The present study selected a total of 158 patients. Among all patients, 63 patients didn't meet the inclusion criteria, 8 patients refused to participate and 7 patients were excluded because the surgery time was longer than 1 h, or the surgery could not be accomplished successfully and turned to laparotomy operation. Finally, 80 elderly patients were included and analyzed. The flow chart is shown in Figure 1. Among the patients, 24 (30%) patients were diagnosed as symptomatic gallstones, 36 (45%) patients were diagnosed as acute or chronic cholecystitis and 20 (25%) patients were diagnosed as biliary pancreatitis. No significant difference was found among different groups of patients (Table 1).

**Table 1:** Basic characteristics of all patients.

Variables	Parecoxib n=20	Dexmedetomidine n=20	Combined n=20	Control n=20
Age, year	67.2±11.4	69.3±12.5	70.4±14.7	68.7±13.5
Gender, male: female	12:8	11:10	13:9	11:6
ASA, n (%)				
I	6 (30)	7 (35)	7 (35)	5 (25)
II	14 (70)	13 (65)	13 (65)	15 (75)
Surgery duration, min	47.5±14.8	49.1±11.2	46.3±10.9	47.4±12.7
Pathological type, n (%)				
Gallstones	8 (40)	5 (25)	6 (30)	5 (25)
Cholecystitis	7 (35)	9 (45)	10 (50)	10 (50)
Biliary pancreatitis	5 (25)	6 (30)	4 (20)	5 (25)

ASA Anesthesiologists grade

### Levels of HR, MAP and SjvO2, PjvO2 in different groups of patients at different time points

As shown in Table 2, the levels of HR, MAP and SjvO2, PjvO2 at different time points were measured and collected. Results showed that no significant difference was found for HR and MAP levels among different groups at all the time points. However, levels of both SjvO2 and PjvO2 were significantly higher in parecoxib group, dexmedetomidine group and the combined group than the control group at T2 and T3 time points (P<0.05). Meanwhile, levels of both SjvO2 and PjvO2 in the combined group were the highest of all other groups at T2 and T3 time points (P<0.05).

**Table 2:** Levels of HR, MAP and SjvO2, PjvO2 in different groups of patients at different time points.

Variables	Groups n=20	T0	T1	T2	T3
HR, frequency/min	Parecoxib	78.3±9.4	79.0±8.7	79.6±9.5	78.3±7.3
	Dexmedetomidine	77.9±8.6	78.2±6.0	81.7±8.8	78.6±7.9
	Combined	78.4±6.1	76.8±8.3	81.4±9.7	79.5±8.7
	Control	79.1±8.9	80.3±7.2	83.9±7.2	81.1±7.2
MAP, mm Hg	Parecoxib	95.3±8.2	87.2±8.5	94.5±8.1	95.7±8.3
	Dexmedetomidine	97.1±9.5	86.1±7.6	95.7±8.3	96.4±7.5
	Combined	96.4±6.7	87.5±7.8	95.6±8.1	95.8±6.9
	Control	96.8±7.4	87.3±8.3	95.3±9.0	96.5±9.3
SjvO2	Parecoxib	60.3±7.1	72.4±9.2	66.3±7.9*,#	64.3±5.6*,#
	Dexmedetomidine	61.2±8.9	73.1±7.1	66.1±5.2*,#	64.2±5.1*,#
	Combined	61.7±6.0	73.9±7.6	71.7±6.4*	68.5±5.7*
	Control	60.9±7.5	72.8±6.3	63.5±5.8	61.0±5.5
PjvO2	Parecoxib	40.7±5.0	48.9±5.4	44.7±4.5*,#	42.5±2.2*,#
	Dexmedetomidine	40.9±6.3	48.2±5.6	44.9±4.2*,#	42.2±2.0*,#
	Combined	41.3±5.8	49.5±5.7	48.3±5.1*	44.3±3.1*
	Control	41.6±5.6	49.7±6.0	41.1±4.9	41.0±1.6

HR heart rate, MAP mean arterial pressure, SjvO2 jugular venous oxygen saturation, PjvO2 jugular venous oxygen pressure.

* P<0.05, compared with the control group;

# P<0.05 compared with the combined group.

### Pain conditions of different groups of patients by VAS scores and PCIA pressing times

Further pain condition analysis showed in patients of parecoxib group, dexmedetomidine group and the combined group, VAS scores were significantly lower than the control group at all the time points except for 48 h after surgery (P<0.05, Table 3). Moreover, VAS scores were significantly lower in the combined group than all other groups except for 48 h after surgery (P<0.05). Similarly, total PCIA pressing times within 48 h after surgery were the lowest in the combined group (P<0.05), while the highest in the control group (P<0.05). These results suggested combined use of parecoxib and dexmedetomidine could significantly reduce the postoperative pain of patients after laparoscopic cholecystectomy.

**Table 3:** Pain conditions of different groups of patients by VAS scores and PCIA pressing times.

Variables	Time points	Parecoxib	Dexmedetomidine	Combined	Control
VAS	4 h	3.7±1.7^c^,^d^	3.9±1.3^c^,^d^	2.4±1.0^a^,^b^,^d^	5.8±2.3^a^,^b^,^c^
	12 h	2.9±0.9^c^,^d^	3.0±1.2^c^,^d^	1.8±0.4^a^,^b^,^d^	4.6±1.7^a^,^b^,^c^
	24 h	1.9±0.2^c^,^d^	2.1±0.6^c^,^d^	1.4±0.1^a^,^b^,^d^	2.8±0.8^a^,^b^,^c^
	48 h	1.1±0.1^d^	1.0±0.1^d^	1.1±0.1 d	1.2±0.1^a^,^b^,^c^
PCIA	48 h	5.0±1.3^c^,^d^	5.1±1.9^c^,^d^	3.4±1.4^a^,^b^,^d^	7.8±2.3^a^,^b^,^c^

a P<0.05, compared with the Parecoxib group;

b P<0.05, compared with DEX group;

c P<0.05, compared with Combined group;

d P<0.05, compared with Control group.

VAS Visual Analogue Score, PICA patient-controlled intravenous analgesia pump.

### Ramsay and MMSE scores and postoperative agitation of different groups of patients after surgery

At last, the postoperative sedation and cognitive conditions were analyzed using Ramsay and MMSE scores, respectively. As shown in Table 4, both Ramsay and MMSE scores were the highest in the combined group compared with other groups (P<0.05), while were the lowest in the control group. These results indicated the combined use of parecoxib and dexmedetomidine could significantly improve the postoperative sedation and cognitive conditions of patients after laparoscopic cholecystectomy.

**Table 4:** Pain conditions of different groups of patients by MMSE and Ramsay scores.

Variables	Time points	Parecoxib	Dexmedetomidine	Combined	Control
MMSE	Before	26.3±1.4	26.5±1.7	27.0±1.5	26.8±1.5
	12 h	22.6±1.6^c^,^d^	22.4±1.9^c^,^d^	24.5±1.6^a^,^b^,^d^	19.4±1.8^a^,^b^,^c^
	24 h	24.0±1.4^c^,^d^	23.7±1.3^c^,^d^	25.7±1.8^a^,^b^,^d^	22.3±1.4^a^,^b^,^c^
	48 h	25.1±1.5^d^	25.0±1.5^d^	26.9±1.9^d^	24.1±1.1^a^,^b^,^c^
Ramsay	12 h	2.4±0.7^c^,^d^	2.5±0.5^c^,^d^	2.8±0.5^a^,^b^,^d^	1.9±0.4^a^,^b^,^c^
	24 h	2.3±0.6^c^,^d^	2.3±0.5^c^,^d^	2.6±0.3^a^,^b^,^d^	2.0±0.6^a^,^b^,^c^
	48 h	2.3±0.5^d^	2.3±0.6^d^	2.5±0.8^d^	2.0±0.3^a^,^b^,^c^

a P<0.05, compared with the Parecoxib group;

b P<0.05, compared with DEX group;

c P<0.05, compared with Combined group;

d P<0.05, compared with Control group.

MMSE mini-mental state examination score.

## Discussion

Though several studies have reported efficacy of parecoxib and dexmedetomidine in cholecystectomy, few of them focused on the combined use of parecoxib and dexmedetomidine. In the present study, we demonstrated that the combined use of parecoxib and dexmedetomidine could reduce postoperative pain and improve postoperative sedation and cognitive conditions of patients after laparoscopic cholecystectomy.

Efficacy of parecoxib in postoperative treatment has been reported in many surgeries, including cholecystectomy. Shuying et al showed intravenous parecoxib could reduce the length of stay on ambulatory for patients who underwent laparoscopic cholecystectomy^16^. Luo *et al*.^17^ demonstrated intravenous injection of parecoxib could provide effective analgesic effect in laparoscopic cholecystectomy and was better than flurbiprofen in terms of analgesic effect. It was also found parecoxib could reduce shoulder pain after gynecologic laparoscopy^18^. In the present study, we also found parecoxib could reduce the postoperative pain and improve the postoperative sedation and cognitive conditions of patients after laparoscopic cholecystectomy, which was consistent with previous researches. And the application of the combined use of dexmedetomidine could enhance the effects.

Application of dexmedetomidine in cholecystectomy has also been demonstrated in several studies. Kang et al showed dexmedetomidine administration during surgery could reduce intraoperative and post-operative secretion of cytokines and post-operative leukocyte count and CRP levels of cholecystectomy patients^19^. Yu *et al*.^20^ demonstrated ropivacaine combined with dexmedetomidine could significantly improve the pain condition for patients after laparoscopic cholecystectomy. It was also considered dexmedetomidine could shift the Th1/Th2 cytokine balance toward Th1 in patients with surgical and anesthetic stress^21^. In our study, we demonstrated the combined use of parecoxib and dexmedetomidine could reduce the postoperative pain and improve the postoperative sedation and cognitive conditions of patients after laparoscopic cholecystectomy, which might provide a new method to improve postoperative recovery. The present study also has some limitations. We included a small sample size from a single hospital and deeper insights as well as more clinical evidences are still needed for the application of parecoxib and dexmedetomidine in postoperative treatment.

## Conclusions

The combined use of parecoxib and dexmedetomidine could reduce the postoperative pain and improve the postoperative sedation and cognitive conditions of patients after laparoscopic cholecystectomy. This study might give more clinical evidence for the application of parecoxib and dexmedetomidine in the postoperative treatment of elderly patients under laparoscopic cholecystectomy.
